# A New Essential Oil from the Native Andean Species *Nectandra laurel* Klotzsch ex Nees of Southern Ecuador: Chemical and Enantioselective Analyses

**DOI:** 10.3390/plants12183331

**Published:** 2023-09-21

**Authors:** Gianluca Gilardoni, Aníbal A. Enríquez, Yessenia E. Maldonado, Nixon Cumbicus, Omar Malagón

**Affiliations:** 1 Departamento de Química, Universidad Técnica Particular de Loja (UTPL), Calle Marcelino Champagnat s/n, Loja 110107, Ecuador or gianluca.gilardoni@gmail.com (G.G.); aaenriquez3@utpl.edu.ec (A.A.E.); yemaldonado2@utpl.edu.ec (Y.E.M.); 2 Facultad de Medicina, Universidad Católica de Cuenca (UCACUE), Av. las Américas y Humboldt, Cuenca 010105, Ecuador; 3 Departamento de Ciencias Biológicas y Agropecuarias, Universidad Técnica Particular de Loja (UTPL), Calle Marcelino Champagnat s/n, Loja 110107, Ecuador; nlcumbicus@utpl.edu.ec

**Keywords:** β-cyclodextrines, enantiomers, gas chromatography, mass spectrometry, *Nectandra mollis*, *Nectandra tovarensis*, *Nectandra willdenoviana*, δ-selinene, sesquiterpenes

## Abstract

The leaves of *Nectandra laurel* Klotzsch ex Nees, belonging to the family, Lauraceae, were collected in the province of Loja (Ecuador), dried, and analytically steam-distilled. An unprecedented essential oil was obtained, with a 0.03% yield by weight of dry plant material. The volatile fraction was submitted to qualitative (GC-MS) and quantitative (GC-FID) chemical analysis, on two orthogonal stationary phases. Seventy-eight compounds were detected and quantified on at least one column. The essential oil was dominated by sesquiterpene hydrocarbons (53.0–53.8% on the non-polar and polar stationary phase, respectively), followed by oxygenated sesquiterpenoids (18.9–19.0%). A third group was constituted by metabolites of other origins, mainly aliphatic compounds, apparently derived from the acetate pathway (11.7–8.5%). The major components of the EO (≥3.0% with at least one column) were δ-selinene (30.5–28.8%), δ-cadinene (5.4–6.4%), *epi*-α-cadinol (4.9–5.2%), an undetermined compound with a molecular weight of 204 (3.4–4.2%), α-pinene (3.3–2.9%), and α-cadinol (2.9–3.0%). Finally, the essential oil was submitted to enantioselective analysis, on two β-cyclodextrin-based chiral selectors, determining the enantiomeric distribution of seven chiral terpenes. Among them, (1*R*,5*R*)-(+)-α-pinene, (1*R*,5*R*)-(+)-β-pinene, and (*R*)-(−)-α-phellandrene were enantiomerically pure, whereas camphene, borneol, α-copaene, and α-terpineol were present as scalemic mixtures.

## 1. Introduction

Located across the equatorial line, Ecuador is a relatively small country of the South American continent. Thanks to its orography and geographic location, it is characterized by the presence of four climatic regions: the Galapagos islands, the Pacific coast, the Andes mountains, and the Amazon Forest. Due to these very diversified climes, Ecuador possesses an extremely high biodiversity, which makes this territory a so-called “megadiverse country” [1]. According to the Catalogue of Vascular Plants of Ecuador, at the date of publication, this country hosted 16,087 botanical species, of which 15,306 were natives and 4173 endemics [2]. So far, from the chemical point of view, most of these native species are completely unstudied or poorly investigated, making Ecuador a potential source of new bioactive molecules and unprecedented natural products [3,4].

On these premises, our group has been investigating Ecuadorian biodiversity for more than twenty years, in search of new or rare secondary metabolites of biological interest [5,6,7]. During the last 6 years, the authors have been focusing on the description of new essential oils (EOs), with an emphasis in the enantiomeric composition, in the characterization of major uncommon sesquiterpenes, and in the olfactometric description of the aroma profile [8,9,10]. Under these premises and with the aim of contributing to the advance in the knowledge on the phytochemistry of Ecuadorian flora, the present work perfectly fits in the described context.

**Figure 1:**
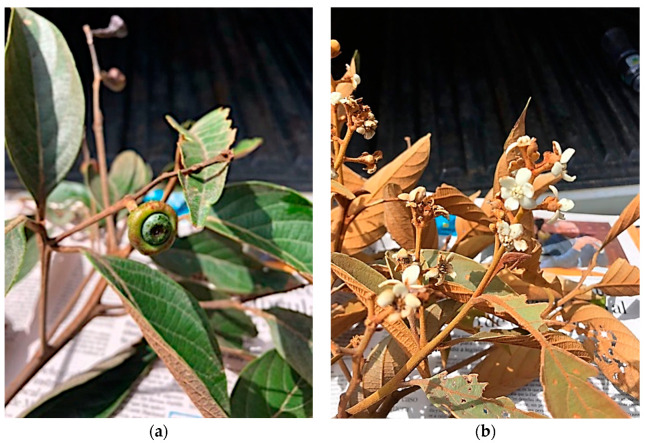
*Nectandra laurel* Klotzsch ex Nees at the collection site; (**a**) fruits and leaves, (**b**) flowers and leaves (Photo by Nixon Cumbicus).

The genus, *Nectandra* Rol. ex Rottb., belonging to the family, Lauraceae, posseses a total of 311 registered species worldwide, of which 101 are accepted [11]. In Ecuador, this genus possesses 36 known species, of which 6 are endemics [2]. The taxon, *Nectandra laurel* Klotzsch ex Nees (see Figure 1), is a tree, native to the Andean region and diffused in Venezuela, Colombia, Ecuador, Perú, and Bolivia [12]. In Ecuador, this species has been described in the provinces of Azuay, Bolívar, Carchi, Chimborazo, Imbabura, Loja, Napo, and Pichincha, where it grows in the range of 1000–3500 m above sea level [2]. Besides this name, this plant is also known by four others: *Nectandra mollis* subsp. *laurel* (Klotzsch ex Nees) Rohwer, *Nectandra tovarensis* Klotzsch & H.Karst. ex Nees, *Nectandra laurel* var. *glabrescens* Meisn., and *Nectandra willdenoviana* Nees [11]. So far, no study has been found in the literature on the phytochemistry of this taxon, either as *N. laurel* or with any of its other synonyms. However, at least 10 other *Nectandra* spp. have been previously studied and had their EOs described. This is the case for *N. amazonum*, *N. barbellata*, *N. cuspidata*, *N. gardneri*, *N. grandiflora*, *N. hihua*, *N. lanceolata*, *N. leucantha*, *N. megapotamica*, and *N. puberula* [13].

The objective of the present research is to describe the chemical and enantiomeric composition of an EO from *Nectandra laurel* Klotzsch ex Nees. that, to the best of the authors’ knowledge, is reported here for the first time.

## 2. Results

### 2.1. Chemical Analysis of N. laurel EO

The dry leaves of *N. laurel* afforded an EO, with a 0.03 ± 0.002% yield by weight, analytically calculated over four repetitions. A total of seventy-eight compounds were detected in the volatile fraction and quantified on at least one column, corresponding to 94.6–91.3% of the oil mass on the non-polar and polar stationary phase, respectively.

**Figure 2:**
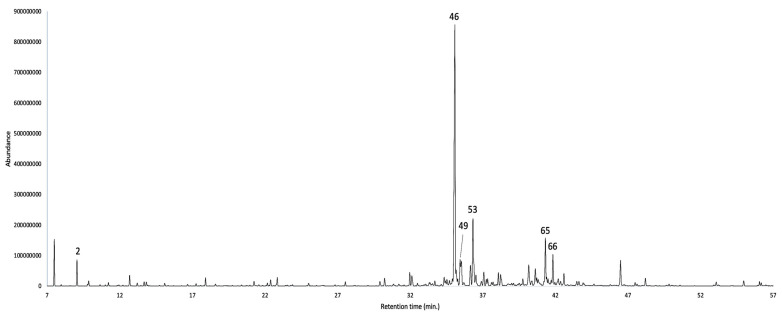
GC-MS profile of *N. laurel* EO on a 5%-phenyl-methylpolysiloxane stationary phase. The peak numbers refer to major compounds (≥3.0% on at least one column), according to Table 1.

**Figure 3:**
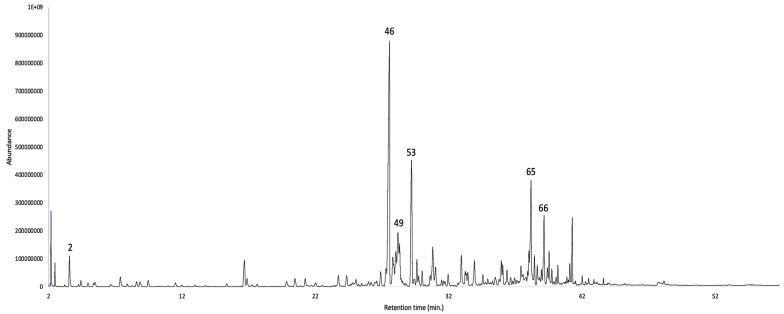
GC-MS profile of *N. laurel* EO on a polyethylene glycol stationary phase. The peak numbers refer to major compounds (≥3.0% on at least one column), according to Table 1.

According to the chemical composition, the EO was dominated by sesquiterpene hydrocarbons (53.0–53.8%), followed by oxygenated sesquiterpenoids (18.9–19.0%). A third main group was constituted by metabolites of other origins, mainly aliphatic compounds, apparently derived from the acetate pathway (11.7–8.5%). The major components of the EO (≥3.0% with at least one column) were δ-selinene (30.5–28.8%), δ-cadinene (5.4–6.4%), *epi*-α-cadinol (4.9–5.2%), an undetermined compound with a molecular weight of 204 (peak 49, 3.4–4.2%), α-pinene (3.3–2.9%), and α-cadinol (2.9–3.0%). The detailed chemical composition is reported in Table 1, whereas the gas chromatography-mass spectrometry (GC-MS) profiles with both columns are represented in Figure 2 and Figure 3.

### 2.2. Enantioselective Analysis of N. laurel EO

The EO from *N. laurel* was subjected to enantioselective analysis, detecting seven chiral compounds whose enantiomers are suitable for separation via at least one of the two applied chiral selectors. Most of the optical isomers were analyzed on a 2,3-diacetyl-6-*tert*-butyldimethylsilyl-β-cyclodextrin stationary phase, whereas the enantiomers of camphene and α-terpineol were evaluated with a 2,3-diethyl-6-*tert*-butyldimethylsilyl-β-cyclodextrin-based column, being inseparable on the other one. On the one hand, (1*R*,5*R*)-(+)-α-pinene, (1*R*,5*R*)-(+)-β-pinene, and (*R*)-(−)-α-phellandrene were found to be enantiomerically pure; on the other hand, camphene, borneol, α-copaene, and α-terpineol were present as scalemic mixtures. The detailed results are reported in Table 2.

## 3. Discussion

As previously described, the EO from leaves of *N. laurel* is dominated by sesquiterpene hydrocarbons (about 50%), followed by oxygenated sesquiterpenoids (about 19%). The sesquiterpene hydrocarbon, δ-selinene, alone constitutes about 30% of the whole oil mass. According to the literature, the EOs from the genus, *Nectandra*, can practically be divided into five main groups: (1) EOs based on monoterpene hydrocarbons, (2) EOs based on sesquiterpene hydrocarbons, (3) EOs based on oxygenated sesquiterpenoids, (4) EOs based on both sesquiterpene hydrocarbons and oxygenated sesquiterpenoids, and (5) EOs based on phenylpropanoids and sesquiterpenes [13]. The EO described in the present study clearly belongs to group (2), including species such as *N. amazonum*, *N. cuspidata*, *N. hihua*, some specimens of *N. megapotamica*, and *N. leucantha*. However, in all these plants, the two major components are usually (*E*)-β-caryophyllene and bicyclogermacrene, with other compounds such as β-selinene, α-humulene, δ-cadinene, and β-bourbonene as other important components. Apparently δ-selinene, our main constituent, is not a major compound in any other known *Nectandra* spp. other than *N. laurel*. Group (1) includes some specimens of *N. megapotamica*, where both pinenes are usually dominants. Group (3) contains *N. grandiflora*, *N. lanceolata*, and other specimens of *N. megapotamica*, where *iso*-bicyclogermacrenal, spathulenol, and α-bisabolol are major constituents. For what concerns group (4), *N. megapotamica* is once again the main representative. Finally, group (5) is represented by *N. puberula*, whose main EO component is apiole [13]. Interestingly, it can be observed that the chemical composition of the EO from different specimens of *N. megapotamica* was so variable that the species could be located in all groups. Based on this phenomenon, it can be hypothesized that *N. megapotamica* EO is just the most studied among the volatile fractions of a very variable genus, and that similar results could also be obtained for *N. laurel*, studying the EO at different times and from different geographical regions. The same literature underlines that chemical variability is a typical feature of Lauraceae, where it is observed more because of seasonal changes than according to the vegetative stage of the plant. This fact is consistent with the need for different insect pheromones in different climes. Other *Nectandra* spp., reported for presenting important seasonal variations, are *N. lanceolata* and *N. grandiflora* [13].

As already mentioned, δ-selinene is absolutely the main component of this EO, reaching about 30% of the whole oil mass. Therefore, not only *N. laurel* EO could be considered as a source of this sesquiterpene, but its biological properties could also theoretically be predicted, at least partially, from the activities of this compound. However, no exhaustive investigations have been found in the literature about the biological activities of pure δ-selinene. Nevertheless, they can be deduced from the properties of other EOs, where this terpene predominates with an amount like the one of *N. laurel*. Three species were identified that produce an EO with these features: *Jatropha elliptica* rhizomes (Euphorbiaceae) from Brazil, *Globba pendula* rhizomes (Zingiberaceae) from Indochina, and *Xanthium italicum* flowers (Asteraceae) from Corsica [59,60,61]. The amount of δ-selinene in the volatile fraction of these plants was 35.7%, 36.4%, and 22.4%, respectively; however, only for *G. pendula* EO were biological activities investigated. In this case, the oil showed a moderate inhibitory capacity on NO production in LPS-activated macrophages, with an IC_50_ = 41.68 ± 4.51% versus 6.51 ± 0.31% of the positive control (NG-methyl-L-arginine acetate) [60]. The same EO also showed a moderate in vitro cytotoxic activity against Hep3B (human hepatoma) and MCF7 (human breast carcinoma) cell lines, with an IC_50_ of 35.24 ± 0.06% and 28.15 ± 1.08%, respectively versus 0.59 ± 0.19% and 6.46 ± 0.81% for the positive control (camptothecin) [60]. Many different biological essays were also carried out on EOs from other *Nectandra* spp., such as *N. amazonum* (anti-leishmanial and cytotoxic), *N. cuspidata* (antibacterial and cytotoxic), *N. gardneri* (anti-leishmanial and cytotoxic), *N. grandiflora* (antibacterial, antifungal, and sedative in silver catfish), *N. hihua* (anti-leishmanial and cytotoxic), *N. lanceolata* (antifungal, antioxidant, anti-chemotactic, cytotoxic, and antibacterial), *N. leucantha* (cytotoxic), *N. megapotamica* (antibacterial, cytotoxic, larvicidal, anaesthetic to some fish species, antifungal, antioxidant, anti-chemotactic, and anti-leishmanial), and *N. puberula* (antibacterial and cytotoxic) [13]. However, due to the different chemical composition of all these EOs compared to the one described here, none of these biological activities can be hypothetically extended to *N. laurel*.

In 2017, Oliveira et al. discovered, in *N. megapotamica* EO, five new oxygenated sesquiterpenoids, denominated nectandrenes. All these metabolites were characterized by the molecular formula, C_15_H_25_O, corresponding to 220 *m*/*z* [62]. In Table 1, it can be observed that, among the undetermined compounds, three sesquiterpenes are isomers of nectandrenes. However, according to data reported in the literature, neither the MS spectra nor the LRIs of these molecules corresponded to any of them.

Finally, the present study was complemented with the enantiomeric composition of some chiral compounds. Ultimately, due to the difficult commercial availability of enantiomerically pure δ-selinene, no chiral information could be obtained from the enantioselective analysis on the major compound. Nevertheless, seven chiral metabolites could be analysed, determining that three of them were enantiomerically pure; one presented a very high enantiomeric excess, whereas three were determined to be scalemic mixtures. As usual, these results demonstrate the existence, in *N. laurel* metabolism, of different enantioselective biosynthetic pathways, devoted to the synthesis of different enantiomers for different functions. It is in fact well known that despite presenting the same physicochemical properties (except the chiroptical ones), two enantiomers can show different biological and physiological activities. It is typical among EOs that two enantiomers present different aromas or different properties as insect pheromones [63,64]. In *N. laurel* EO, the only major constituent (≥3.0% on at least one column), whose stereochemistry could be determined, was the enantiomerically pure (1*R*,5*R*)-(+)-α-pinene. According the to literature, dextrorotatory α-pinene has been demonstrated to be an antibacterial, antimalarial, and anti-inflammatory agent. Furthermore, it was much more active as an antimycotic and anticatabolic agent compared to the laevorotatory isomer. Finally, both optical isomers are known for being active as acetylcholinesterase inhibitors [65].

## 4. Materials and Methods

### 4.1. Plant Material

The leaves of *N. laurel* were collected on 26 November 2020 (unintentional date), with the permission of the Ministry of Environment, Water, and Ecological Transition of Ecuador, with MAATE registry number MAE-DNB-CM-2016-0048. The collection site was located in the radius of 200 m from a central point, of coordinates 04°22′46′′ S and 79°08′46′′ W, at an altitude of 2350 m above sea level. The botanical identification was carried out by one of the authors (N.C.), based on collection reviews conserved at the herbarium of the Universidad Nacional de Loja (UNL), Ecuador. A botanical specimen was also deposited at the herbarium of the Universidad Técnica Particular de Loja with code 14,702.

On the day of collection, the fresh leaves were dried at 35 °C for 48 h and the dry plant material (355 g) was stored in a fresh dry place until use.

### 4.2. EO Distillation and Sample Preparation

The dry leaves were analytically steam-distilled in a Marcusson-type apparatus, as previously described in the literature [8]. The process was repeated four times, on amounts of 80 g for 4 h. The distillation was conducted on 2 mL of cyclohexane, spiked with *n*-nonane as internal standard (0.71 mg/mL), producing four samples of EO in solution that could directly be injected into GC. Both cyclohexane and *n*-nonane were analytical purity grade and purchased from Signa-Aldrich (Saint Louis, MO, USA). During the entire investigation, the samples were always stored in the dark at −15 °C until use.

### 4.3. Qualitative (GC-MS) Chemical Analysis of N. laurel EO

The qualitative chemical analysis of the EO was conducted in a Trace 1310 gas chromatograph (GC), coupled with a ISQ 7000 mass spectrometer (MS) as a detector (Thermo Fisher Scientific, Walthan, MA, USA). The oven was equipped with a non-polar DB-5ms and a polar HP-INNOWax column, both 30 m long, 0.25 mm internal diameter, and 0.25 μm film thickness (Agilent Technology, Santa Clara, CA, USA). With both columns, the following thermal program was applied: 50 °C for 10 min., followed by a first gradient of 3 °C/min until 100 °C, a second gradient of 5 °C/min until 200 °C, and finally, a third gradient of 10 °C/min until 230 °C, that were maintained for 20 min. The injector and transfer line were maintained at 230 °C, with the injector operating in split mode (40:1) and the autosampler injecting 1 μL. The carrier gas was helium, flowing through the column at the constant flow of 1 mL/min, and purchased from Indura (Guayaquil, Ecuador). The MS was operated in SCAN mode, with a mass range of 40–400 *m*/*z*; the electron impact (EI) ion source was set at 70 eV and 250 °C. All the EO constituents were identified, on both columns, by comparing each mass spectrum and linear retention index (LRI) with data from the literature. The LRIs were calculated based on the retention times of a mixture of homologous *n*-alkanes (C_9_-C_22_ from Sigma-Aldrich, Saint Louis, MO, USA), injected via the same GC method according to Van den Dool and Kratz [66].

### 4.4. Quantitative (GC-FID) Chemical Analysis of N. laurel EO

The quantitative chemical analysis was carried out with the same GC, columns, thermal program, and instrument configuration used for the qualitative one but with the exception of the detector, that was in this case, a FID (flame ionization detector), set to 250 °C. The EO components were quantified calculating each relative response factor (RRF) according to the corresponding combustion enthalpies [67,68]. The transformed integration areas were applied to two six-point calibration curves (one for each column), obtaining two correlation coefficients greater than 0.995 [69]. Isopropyl caproate was synthetized in one of the authors’ laboratories (G.G.) and purified until 98.8% (GC purity).

### 4.5. Enantioselective GC-MS Analysis of N. laurel EO

The enantioselective analysis was performed via GC-MS, with the same instrument and MS configuration of the qualitative chemical analysis. However, in this case, the elution was conducted at the constant pressure of 70 kPa. The oven was equipped with two enantioselective columns, whose stationary phases were based on 2,3-diacetyl-6-*tert*-butyldimethylsilyl-β-cyclodextrin and 2,3-diethyl-6-*tert*-butyldimethylsilyl-β-cyclodextrin, respectively. Both columns were purchased from MEGA S.r.l., Legnano, Italy. The analyses were carried out with the following thermal program: 50 °C for 1 min, followed by a thermal gradient of 2 °C/min until 220 °C, that was maintained for 10 min. The enantiomers were identified through comparison of the MS spectra and linear retention indices with data obtained from the injection of enantiomerically pure standards.

## 5. Conclusions

The leaves of *Nectandra laurel* Klotzsch ex Nees produce an essential oil, with a distillation yield of 0.03% by weight of dry plant. Despite the quite low yield, this volatile fraction is mainly composed of δ-selinene, which constitutes one third of the whole oil mass. According to the enantioselective analysis, the enantiomerically pure (1*R*,5*R*)-(+)-α-pinene represents about 3% of the oil mass. This enantiomer is known for being an antibacterial, antimalarial, and anti-inflammatory agent.

## Figures and Tables

**Table 1:** Qualitative (GC-MS) and quantitative (GC-FID) analyses of *N. laurel* EO with two orthogonal stationary phase columns.

N.	Identification	5%-Phenyl-Methylpolysiloxane					Polyethylene Glycol				
		LRI ^a^	LRI ^b^	%	σ	Reference	LRI ^a^	LRI ^b^	%	σ	Reference
1	heptanal	916	901	0.1	0.03	[14]	1181	1182	0.3	0.02	[15]
2	α-pinene	937	932	3.3	0.32	[14]	1015	1015	2.9	0.04	[16]
3	camphene	953	946	0.8	0.04	[14]	1046	1046	0.3	0.03	[16]
4	2-(*E*)-heptenal	969	947	0.4	0.05	[14]	1317	1318	0.4	0.02	[17]
5	benzaldehyde	976	952	0.3	0.05	[14]	1513	1513	0.4	0.02	[18]
6	β-pinene	981	974	0.5	0.04	[14]	1102	1100	0.5	0.03	[19]
7	decane	1000	1000	0.1	0.01	-	1000	1000	0.2	0.03	-
8	α-phellandrene	1010	1002	1.4	0.09	[14]	1156	1158	1.2	0.03	[20]
9	α-terpinene	1020	1014	0.3	0.03	[14]	1171	1167	0.3	0.02	[21]
10	*o*-cymene	1029	1022	0.4	0.02	[14]	1262	1261	0.4	0.02	[22]
11	sylvestrene	1032	1025	0.8	0.05	[14]	1190	1200	0.5	0.02	[23]
12	β-phellandrene	1034	1025	0.1	0.01	[14]	1201	1203	0.2	0.03	[24]
13	(*E*)-β-ocimene	1050	1044	trace	-	[14]	1248	1245	0.1	0.03	[25]
14	phenylacetaldehyde	1057	1036	0.6	0.04	[14]	1637	1638	0.4	0.02	[26]
15	γ-terpinene	1062	1054	0.1	0.02	[14]	1237	1231	0.1	0.03	[27]
16	2-(*E*)-octen-1-al	1069	1049	0.1	0.01	[14]	1421	1423	0.2	0.02	[28]
17	terpinolene	1089	1086	0.2	0.02	[14]	1273	1271	0.2	0.03	[29]
18	*p*-cymenene	1097	1089	0.1	0.01	[14]	1430	1431	0.1	0.03	[30]
19	undecane	1100	1100	0.5	0.03	-	1100	1100	0.4	0.02	-
20	linalool	1107	1095	trace	-	[14]	1553	1549	0.2	0.03	[31]
21	nonanal	1113	1100	1.7	0.1	[14]	1381	1380	1.3	0.03	[16]
22	*exo*-fenchol	1127	1118	0.3	0.03	[14]	-	-	-	-	-
23	camphene hydrate	1163	1145	0.1	0.01	[14]	1588	1602	0.2	0.03	[32]
24	2-(*E*)-nonen-1-al	1171	1157	0.2	0.01	[14]	1529	1526	0.2	0.03	[28]
25	borneol	1181	1165	0.3	0.04	[14]	1695	1693	0.5	0.04	[24]
26	dodecane	1200	1200	0.8	0.04	-	1200	1200	0.6	0.02	-
27	α-terpineol	1205	1186	0.7	0.03	[14]	1692	1692	0.5	0.04	[33]
28	decanal	1215	1201	1.1	0.72	[14]	1492	1494	0.8	0.01	[34]
29	thymol methyl ether	1237	1232	0.1	0.01	[14]	1591	1593	0.2	0.03	[35]
30	undetermined (MW: 152)	1261	-	0.6	0.03	-	1662	-	0.6	0.02	-
31	piperitone	1266	1249	0.1	0.01	[14]	-	-	-	-	-
32	nonanoic acid	1282	1267	0.5	0.2	[14]	2213	2202	0.4	0.04	[36]
33	tridecane	1300	1300	0.1	0.02	-	1300	1300	0.3	0.03	-
34	carvacrol	1312	1298	0.3	0.02	[14]	2214	2215	0.3	0.04	[37]
35	undecanal	1316	1305	0.8	0.02	[14]	1599	1598	0.7	0.02	[38]
36	(2*E*,4*E*)-decadienal	1331	1315	0.1	0.01	[14]	1801	1800	0.1	0.03	[39]
37	α-ylangene	1370	1373	0.5	0.01	[14]	1468	1470	0.1	0.04	[20]
38	α-copaene	1378	1374	0.7	0.02	[14]	1475	1475	0.7	0.02	[24]
39	dodecanal	1418	1408	2.1	0.07	[14]	1706	1708	overlapped to peak 49		[40]
40	undetermined (MW: 204)	1419	-	1.2	0.04	-	1562		0.8	0.02	-
41	(E)-β-caryophyllene	1419	1417			[14]	1582	1580	0.4	0.02	[20]
42	α-(*E*)-ionone	1430	1428	0.4	0.01	[14]	1841	1839	0.5	0.03	[41]
43	β-gurjunene	1432	1431	0.2	0.01	[14]	1668	1655	0.1	0.03	[42]
44	*cis*-cadina-1(6),4-diene	1462	1461	0.3	0.01	[14]	1465	-	0.7	0.03	-
45	γ-muurolene	1479	1478	0.8	0.05	[14]	1676	1678	0.6	0.01	[43]
46	δ-selinene	1492	1492	30.5	0.15	[14]	1739	1728	28.8	0.75	[44]
47	β-selinene	1494	1489	0.4	0.02	[14]	1702	1702	0.4	0.05	[16]
48	valencene	1497	1496	0.4	0.02	[14]	1717	1717	0.2	0.02	[45]
49	undetermined (MW: 204)	1501	-	3.4	0.04	-	1708	-	4.2	0.09	-
50	α-muurolene	1503	1500	2.3	0.05	[14]	1713	1723	1.7	0.07	[32]
51	δ-amorphene	1507	1511	0.5	0.01	[14]	1704	1710	0.4	0.05	[46]
52	y-cadinene	1519	1513	1.5	0.03	[14]	1717	1720	1.2	0.01	[47]
53	δ-cadinene	1523	1522	5.4	0.31	[14]	1747	1747	6.4	0.14	[48]
54	7-*epi*-α-selinene	1525	1520	2.0	2.08	[14]	1764	1762	2.4	0.03	[49]
55	*cis*-calamenene	1528	1528	1.1	0.11	[14]	1821	1835	1.1	0.01	[32]
56	α-calacorene	1549	1544	0.3	0.01	[14]	1902	1904	0.9	0.01	[50]
57	undetermined (MW: 204)	1568	-	1.1	0.01	-	1811	-	2.2	0.03	-
58	undetermined (MW: 220)	1572	-	1.2	0.07	-	1906	-	0.9	0.36	-
59	palustrol	1577	1567	trace	-	[14]	1914	1914	0.1	0.03	[51]
60	gleenol	1594	1586	0.4	0.01	[14]	2029	2032	0.6	0.02	[52]
61	guaiol	1604	1600	0.4	0.07	[14]	2083	2080	0.8	0.02	[53]
62	undetermined (MW: 220)	1611	-	0.9	0.06	-	1980	-	0.3	0.02	-
63	undetermined (MW: 202)	1622	-	1.3	0.08	[14]	1951	-	1.4	0.01	-
64	undetermined (MW: 220)	1634	-	1.9	0.12	-	2158	-	1.5	0.05	-
65	*epi*-α-cadinol	1652	1638	4.9	0.34	[14]	2167	2166	5.2	0.08	[54]
66	α-cadinol	1666	1652	2.9	0.27	[14]	2221	2221	3.0	0.02	[55]
67	cyperotundone	1686	1695	1.3	0.06	[14]	2163	-	1.1	0.05	-
68	*epi*-cyclocolorenone	1795	1774	2.3	0.24	[14]	2338	-	2.4	0.08	-
69	undetermined (MW: 268)	1844	-	1.3	0.14	-	2125	-	1.7	0.12	-
70	nonadecane	1900	1900	0.2	0.02	-	1900	1900	0.1	0.03	-
71	(5*E*,9*E*)-farnesyl acetone	1917	1913	0.1	0.02	[14]	-	-	-	-	-
72	1-eicosene	1993	1987	0.5	0.06	[14]	2048	2047	0.6	0.03	[56]
73	eicosane	2000	2000	0.1	0.02	-	2000	2000	0.2	0.03	-
74	undetermined (MW: 272)	2058	-	0.5	0.06	-	2328	-	0.7	0.03	-
75	1-octadecanol	2095	2090	0.5	0.07	[57]	2572	2570	0.1	0.01	[58]
76	heneicosane	2100	2100	0.3	0.04	-	2100	2100	0.6	0.02	-
77	1-docosene	2195	2189	0.6	0.08	[14]	-	-	-	-	-
78	docosane	2200	2200	trace	0.02	-	2200	2200	0.2	0.03	-
	monoterpene hydrocarbons			8.0					6.8		
	oxygenated monoterpenoids			2.5					2.5		
	sesquiterpene hydrocarbons			53.0					53.8		
	oxygenated sesquiterpenoids			18.9					19.0		
	diterpene hydrocarbons			0.5					0.7		
	others			11.7					8.5		
	total			94.6					91.3		

^a^ Calculated linear retention index; ^b^ reference linear retention index; % = percent amount by weight; σ = standard deviation; MW = molecular weight.

**Table 2:** Enantioselective analysis of *N. laurel* EO on two β-cyclodextrin-based chiral selectors.

Enantiomers	LRI	Enantiomeric Distribution (%)	*e.e*. (%)
(1*R*,5*R*)-(+)-α-pinene	925 *	100.0	100.0
(1*R*,4*S*)-(-)-camphene	960 **	70.3	40.6
(1*S*,4*R*)-(+)-camphene	963 **	29.7	
(1*R*,5*R*)-(+)-β-pinene	978 *	100.0	100.0
(*R*)-(−)-α-phellandrene	1024 *	100.0	100.0
(1*R*,2*S*,4*R*)-(+)-borneol	1297 *	7.8	84.4
(1*S*,2*R*,4*S*)-(-)-borneol	1302 *	92.2	
(1*R*,2*S*,6*S*,7*S*,8*S*)-(−)-α-copaene	1376 *	0.6	98.8
(1*S*,2*R*,6*R*,7*R*,8*R*)-(+)-α-copaene	1380 *	99.4	
(*S*)-(−)-α-terpineol	1400 **	85.5	71.0
(*R*)-(+)-α-terpineol	1405 **	14.5	

LRI = linear retention index; *e.e.* = enantiomeric excess; * 2,3-diacetyl-6-*tert*-butyldimethylsilyl-β-cyclodextrin; ** 2,3-diethyl-6-*tert*-butyldimethylsilyl-β-cyclodextrin.

## Data Availability

Raw data are available from the author (A.A.E.).
